# Deep Vein Thrombosis in an 11-Year-Old Child With Pulmonary Tuberculosis: A Case Report

**DOI:** 10.7759/cureus.112725

**Published:** 2026-07-15

**Authors:** Zineb Benhdech, Azzeddine Laaraje, Abdelilah Radi, Amale Hassani, Rachid Abilkassem

**Affiliations:** 1 Department of Pediatrics, Mohammed V Military Hospital, Rabat, MAR

**Keywords:** deep vein thrombosis, hypercoagulability, pediatric, rifampicin, tuberculosis, venous thromboembolism

## Abstract

Tuberculosis (TB) induces a systemic inflammatory response that can increase thrombotic risk, making venous thrombosis an uncommon but significant complication, especially in children. We present the case of an 11-year-old girl undergoing treatment for pleuro-pulmonary TB who developed acute deep vein thrombosis of the lower limb early during therapy. The diagnosis was established through clinical assessment and imaging, and the patient responded well to anticoagulation while continuing her TB treatment. This case underscores the need for clinicians to remain vigilant for thrombotic complications in pediatric TB, as early identification and management are essential to prevent morbidity.

## Introduction

Tuberculosis (TB) remains, despite medical advances, one of the leading infectious causes of morbidity and mortality worldwide, particularly in low- and middle-income countries such as Morocco [1]. In children, TB represents a significant health burden due to its diverse clinical manifestations, which may delay diagnosis and complicate management. While pulmonary and extrapulmonary forms are well characterized, thromboembolic events remain exceptionally rare and underrecognized in the pediatric population [2]. Patients with TB have been reported to have a four- to eight-fold increased risk of venous thromboembolism (VTE) compared with the general population, although pediatric cases remain exceedingly uncommon [3].

The association between TB and VTE is increasingly recognized, as the infection creates a pro-inflammatory and prothrombotic milieu. This relationship can be explained by Virchow’s triad, which describes the three principal mechanisms promoting thrombosis: hypercoagulability, blood stasis, and endothelial injury. In TB, hypercoagulability is promoted by elevated fibrinogen levels, inflammatory cytokines, thrombocytosis, and the potential prothrombotic effects of anti-TB treatment, particularly rifampicin. Blood stasis may result from prolonged immobilization or venous compression by enlarged lymph nodes, whereas endothelial injury is driven by systemic inflammation and immune activation [4].

This pathological association presents important diagnostic and therapeutic challenges. The nonspecific clinical presentation may delay recognition of thromboembolic complications. In addition, anticoagulant therapy may be complicated by the interaction between rifampicin, a potent inducer of cytochrome P450 enzymes and P-glycoprotein, and both vitamin K antagonists and direct oral anticoagulants, including rivaroxaban, potentially reducing their anticoagulant effect [2,4].

Although these mechanisms are well described in adults, pediatric data remain limited, with only isolated cases of TB-associated deep vein thrombosis (DVT) reported in the literature.

We report the case of an 11-year-old girl with pleuro-pulmonary TB complicated by femoro-popliteal DVT. This case highlights the importance of maintaining a high index of suspicion for thrombotic complications in children with TB and underscores the need for early recognition and prompt management.

## Case presentation

An 11-year-old girl with up-to-date immunizations and normal psychomotor and growth development (weight: 35 kg) presented to the emergency department with a two-week history of fever, cough, and progressive asthenia. She had no known exposure to TB and no personal or family history of venous thromboembolism or thrombophilia.

Initial outpatient treatment with antitussive medication was ineffective. During the following week, she developed worsening fatigue and mild dyspnea, prompting hospital admission.

On admission, she was conscious, febrile (39°C), and hemodynamically stable (blood pressure: 105/65 mmHg; heart rate: 110 beats/min), with an oxygen saturation of 96% on room air. There were no signs of dehydration or peripheral hypoperfusion. General physical examination was otherwise unremarkable. Respiratory examination revealed suprasternal retractions, bilateral coarse crackles, and markedly decreased breath sounds over the right lung base. No thoracic deformity or digital clubbing was observed. Cardiovascular and abdominal examinations were unremarkable. Neurological examination showed normal motor and sensory function, symmetrical deep tendon reflexes, and no focal neurological deficits. A single 1-cm left cervical lymph node was palpable, whereas the remaining lymph node areas were normal. Skin and mucous membrane examination was unremarkable.

Laboratory investigations showed normal hemoglobin, platelet count, and total leukocyte count, with neutrophil predominance, together with an elevated C-reactive protein level. The principal laboratory findings obtained at admission, before initiation of anti-TB therapy and anticoagulation, are summarized in Table 1.

**Table 1:** Laboratory findings on admission

Test	Result	Reference range
Hemoglobin	12 g/dL	11.5–15.5 g/dL
White blood cells	8,000 /mm³	4,000–11,000 /mm³
Neutrophils	6,400 /mm³	1,500–7,500 /mm³
Lymphocytes	1,000 /mm³	1,000–4,000 /mm³
Platelets	232,000 /mm³	150,000–450,000 /mm³
Prothrombin time	77%	70–100%
Activated partial thromboplastin time ratio	1.1	0.8–1.2 (ratio)
C-reactive protein (CRP)	109 mg/L	<5 mg/L
Urea	0.19 g/L	0.13–0.45 g/L
Creatinine	5 mg/L	6–12 mg/L

**Figure 1:**
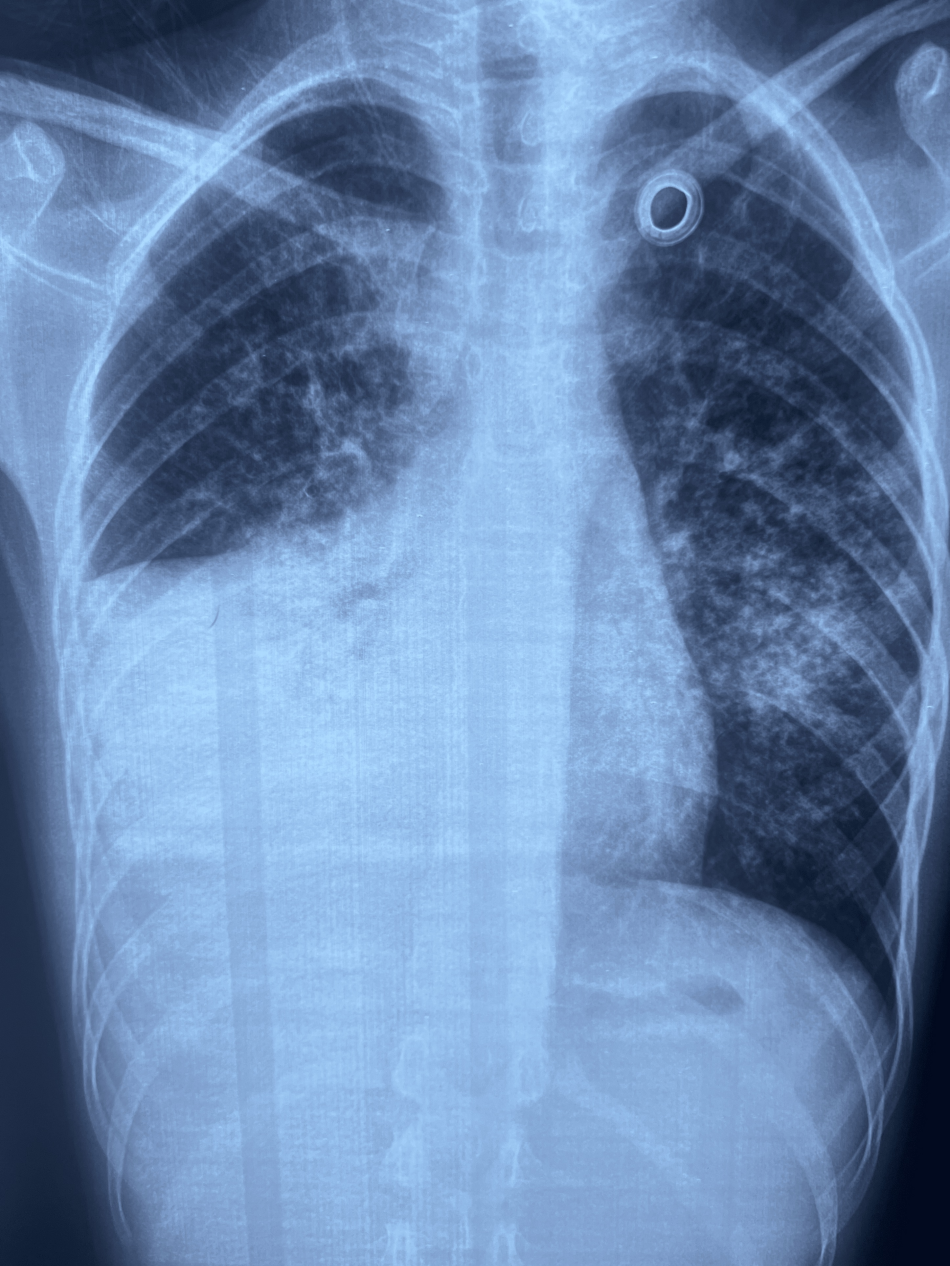
Chest X-ray showing a right pleural effusion, miliary pattern

Chest radiography demonstrated a right-sided pleural effusion with radiological features suggestive of miliary TB (Figure 1).

**Figure 2:**
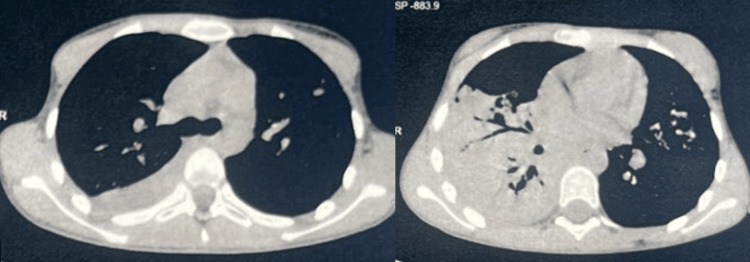
Axial CT slice at the thoracic level in the mediastinal window, without contrast injection, demonstrating a right-sided pleural effusion of low to moderate volume, associated with a lower-lobe pulmonary parenchymal consolidation containing multiple air bronchograms

**Figure 3:**
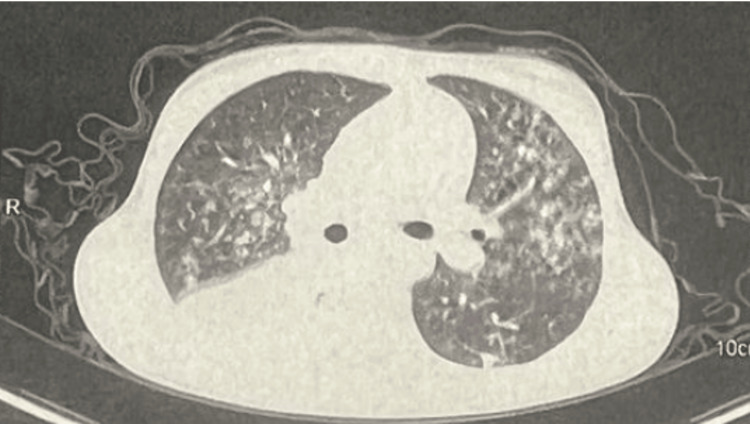
Axial CT slice through the thoracic level in the lung window showing multiple bilateral upper-lobe pulmonary micronodules with a perilymphatic distribution

Thoracic ultrasound demonstrated a minimal, non-puncturable pleural effusion, while chest computed tomography showed findings consistent with infectious pneumonia (Figures 2, 3).

Sputum GeneXpert testing was positive for Mycobacterium tuberculosis, with no rifampicin resistance detected. Lumbar puncture revealed normal biochemical findings, negative bacteriological examination, and a negative search for M. tuberculosis. Abdominal ultrasonography was unremarkable.

Based on these findings, standard anti-TB therapy was initiated with isoniazid (4.4 mg/kg/day), rifampicin (8.8 mg/kg/day), pyrazinamide (23.5 mg/kg/day), and ethambutol (16.1 mg/kg/day) during the intensive phase, followed by isoniazid and rifampicin during the continuation phase, for a total treatment duration of six months.

**Figure 4:**
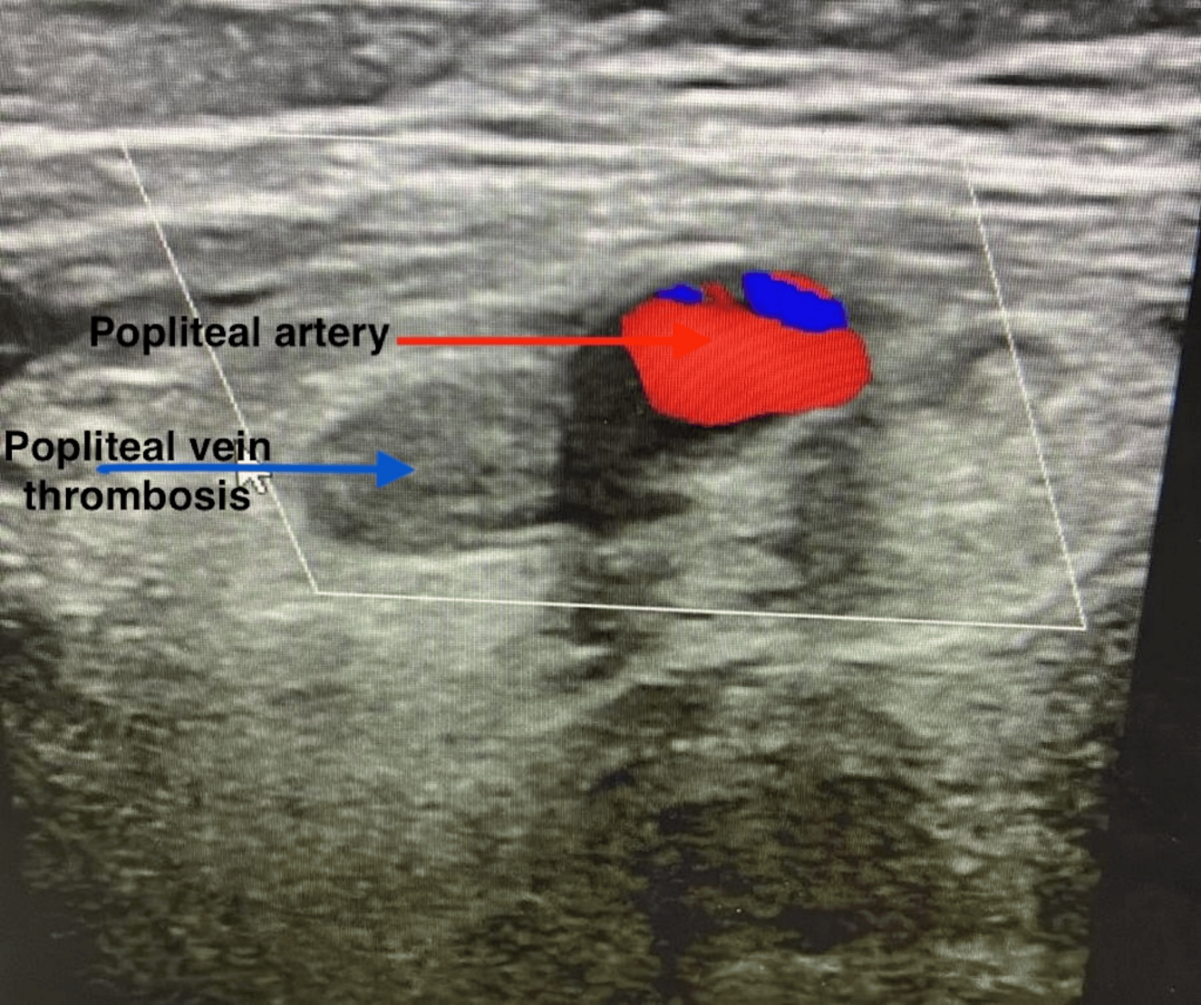
Axial view showing a dilated popliteal vein containing dense material, with no color Doppler flow encoding

**Figure 5:**
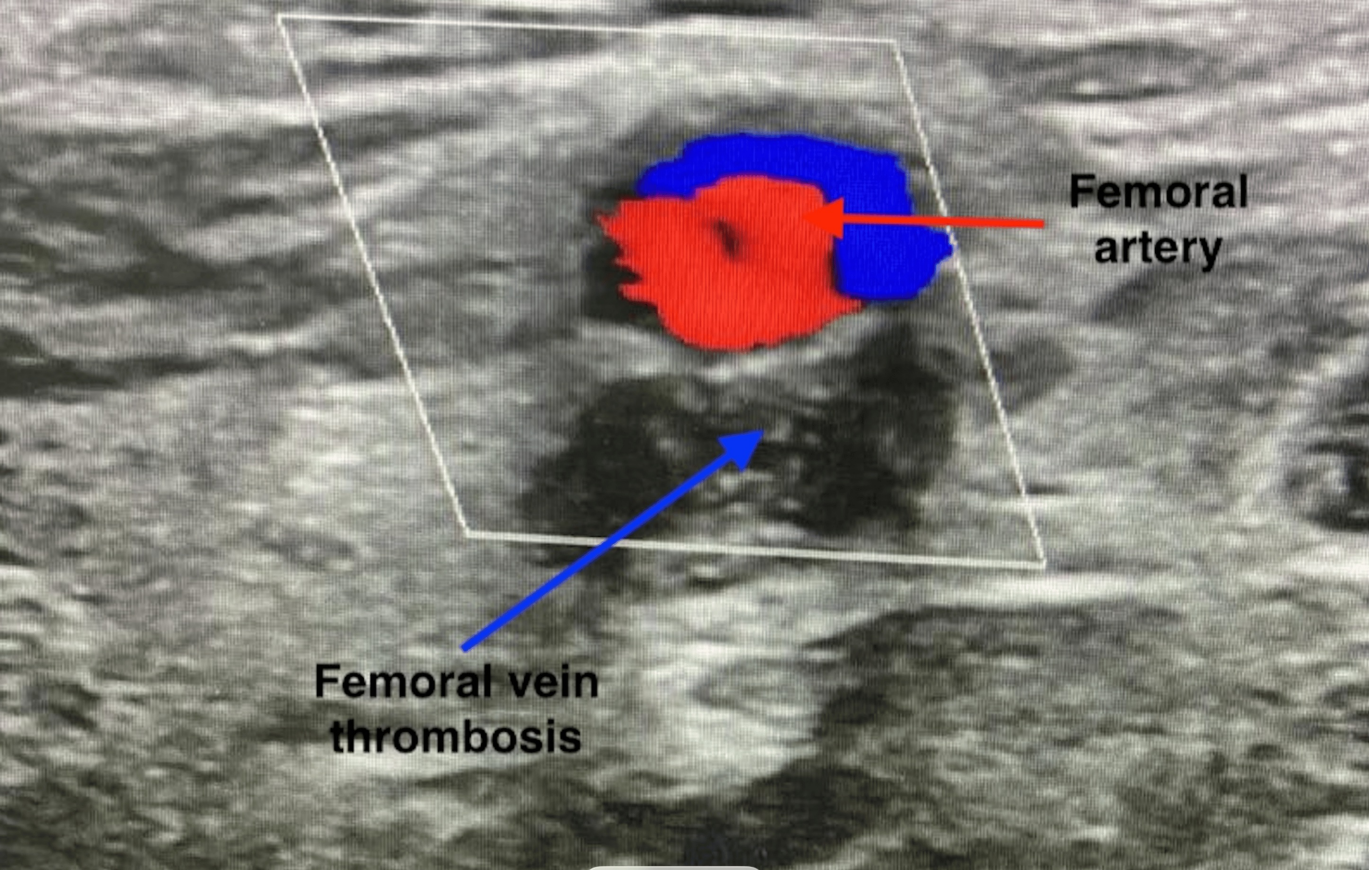
Axial view showing a dilated femoral vein containing dense material, with no color Doppler flow encoding

One week after initiation of anti-TB therapy, the patient progressively developed swelling and pain of the left lower limb. Physical examination revealed a swollen, erythematous calf. D-dimer was markedly elevated at 16,200 ng/mL, prompting Doppler ultrasonography, which confirmed left femoro-popliteal DVT (Figures 4, 5). Transthoracic echocardiography was normal. Baseline coagulation studies, including prothrombin time and activated partial thromboplastin time ratio, were normal. Thrombophilia screening, including protein C, protein S, antithrombin III, coagulation factor assays, lupus anticoagulant, and antiphospholipid antibodies, was unremarkable.

Therapeutic anticoagulation with low-molecular-weight heparin (enoxaparin) was administered for 10 days, followed by rivaroxaban 15 mg once daily for three months.

A repeat Doppler ultrasound performed after three months confirmed complete resolution of the left femoro-popliteal DVT, and rivaroxaban was discontinued. The patient continued anti-TB therapy without interruption. At completion of the six-month anti-TB treatment, she showed a favorable clinical outcome, and anti-TB therapy was discontinued.

## Discussion

TB remains the leading cause of death from a single infectious agent, with over 10 million new cases annually and a persistently high global burden [1]. Its complications are diverse and may involve multiple organ systems, including respiratory, cardiac, neurological, digestive, renal, and hematological manifestations [5].

The risk of VTE is significantly increased in patients with TB compared to the general population, with studies reporting a 4-8-fold higher incidence [6,7]. Early autopsy series have also demonstrated venous thrombosis in up to 24.3% of TB cases [8], although pediatric presentations remain extremely rare.

TB-associated thrombosis is explained by Virchow’s triad. Hypercoagulability results from activation of mononuclear cells by M. tuberculosis, leading to increased production of IL-6, IL-1, and TNF-α, which contribute to endothelial injury [9]. IL-6 further stimulates fibrinogen and C4b-binding protein synthesis, enhancing coagulation pathways [10,11], while thrombocytosis further amplifies the prothrombotic state. Blood stasis may occur due to prolonged bed rest during hospitalization and can be exacerbated by venous compression secondary to lymphadenopathy [12]. Endothelial injury results from cytokine-mediated inflammation and direct vascular effects of TB bacilli, and anti-TB therapy, particularly rifampicin, may further increase thrombotic risk [13].

Thromboembolic events typically occur after the diagnosis of TB but may occasionally represent an initial manifestation of the disease [14]. In our patient, thrombophilia screening, including protein C, protein S, antithrombin III, coagulation factor assays, lupus anticoagulant, and antiphospholipid antibodies, was unremarkable, making TB the most likely precipitating factor.

Low-molecular-weight heparin remains the first-line anticoagulant therapy in pediatric VTE [15,16]. More recently, direct oral anticoagulants such as rivaroxaban and dabigatran have emerged as alternative options in children, with weight-adjusted dosing and favorable safety profiles [17,18]. The rivaroxaban dose used in our patient (15 mg once daily) was consistent with the European Medicines Agency recommendations for pediatric patients weighing less than 50 kg [18]. However, concomitant administration of rifampicin deserves particular attention because rifampicin is a potent inducer of cytochrome P450 3A4 and P-glycoprotein, potentially reducing rivaroxaban plasma concentrations and anticoagulant efficacy [19]. Despite this potential interaction, our patient achieved complete clinical and radiological resolution after three months of anticoagulation, with no recurrence during follow-up. Nevertheless, this combination should be used cautiously, and additional pediatric studies are needed to better define optimal anticoagulation strategies in children receiving rifampicin.

Published pediatric cases of TB-associated DVT remain scarce. Most reported patients developed thrombosis during active pulmonary TB and were successfully treated with anticoagulation combined with anti-TB therapy. Similar to our patient, favorable outcomes have generally been reported when thrombotic complications are recognized promptly and managed appropriately, although evidence remains limited to isolated case reports and small case series. Although the chest CT findings in our patient were initially interpreted as consistent with infectious pneumonia, the diagnosis of pulmonary TB was established based on the overall clinical presentation, chest radiographic findings suggestive of miliary TB, and microbiological confirmation by GeneXpert.

This case emphasizes the importance of early recognition of DVT in children with TB, particularly in the presence of limb swelling or unexplained pain, and the need for prompt anticoagulation while continuing anti-TB therapy. Although prophylactic anticoagulation may be considered in selected high-risk pediatric patients, current evidence remains limited and further studies are required.

## Conclusions

This case demonstrates that DVT can occur in children with pulmonary TB, although this complication remains rare. Early recognition of thrombotic manifestations, prompt initiation of anticoagulation, and continuation of appropriate anti-TB therapy were associated with a favorable clinical outcome in our patient. Given the limited evidence available in pediatric populations, the true incidence, risk factors, and optimal management of TB-associated VTE remain to be established. Clinicians should maintain a high index of suspicion for thrombotic complications in children with TB who present with limb swelling or unexplained pain.

## References

[1] (2024). Global Tuberculosis Report. https://iris.who.int/server/api/core/bitstreams/7292c91e-ffb0-4cef-ac39-0200f06961ea/content.

[2] Hadjadj-Aoul M (2015). Tuberculose et accidents thromboemboliques. Rev Mal Respir.

[3] Sharif-Kashani B, Bikdeli B, Moradi A (2010). Coexisting venous thromboembolism in patients with tuberculosis. Thromb Res.

[4] Ben Amar J, Dahri B, Aouina H, Bouacha H (2015). Venous thromboembolism in patients with acute tuberculosis (Article in French). Rev Pneumol Clin.

[5] Underner M, Perriot J (2013). Complications of disseminated tuberculosis (Article in French). Rev Mal Respir.

[6] Wendelboe AM, Raskob GE (2016). Global burden of thrombosis: epidemiologic aspects. Circ Res.

[7] Purayil NK, Sirajudeen J, Al Arbi KM, Baghi MA, Zahid M (2021). Venous thromboembolism: an unusual presentation of pulmonary tuberculosis. Cureus.

[8] Moran TJ (1950). Autopsy incidence of pulmonary embolism in tuberculosis. Dis Chest.

[9] Law K, Weiden M, Harkin T, Tchou-Wong K, Chi C, Rom WN (1996). Increased release of interleukin-1 beta, interleukin-6, and tumor necrosis factor-alpha by bronchoalveolar cells lavaged from involved sites in pulmonary tuberculosis. Am J Respir Crit Care Med.

[10] Turken O, Kunter E, Sezer M (2002). Hemostatic changes in active pulmonary tuberculosis. Int J Tuberc Lung Dis.

[11] Koster T, Vandenbroucke JP, Rosendaal FR, de Ronde H, Briet E, Bertina RM (1993). Venous thrombosis due to poor anticoagulant response to activated protein C: Leiden thrombophilia study. Lancet.

[12] Robson SC, White NW, Aronson I, Woollgar R, Goodman H, Jacobs P (1996). Acute-phase response and the hypercoagulable state in pulmonary tuberculosis. Br J Haematol.

[13] White NW (1989). Venous thrombosis and rifampicin. Lancet.

[14] Dakouo MR, Samake S, Sogodogo A (2025). Venous thromboembolic disease revealing multifocal tuberculosis: a case report. Health Res Afr.

[15] Newall F, Branchford B, Male C (2018). Anticoagulant prophylaxis and therapy in children: current challenges and emerging issues. J Thromb Haemost.

[16] Klaassen IL, Sol JJ, Suijker MH, Fijnvandraat K, van de Wetering MD, Heleen van Ommen C (2019). Are low-molecular-weight heparins safe and effective in children? A systematic review. Blood Rev.

[17] Male C (2022). Anticoagulation in pediatric patients. Hamostaseologie.

[18] (2026). European Medicines Agency. Xarelto product information. https://www.ema.europa.eu/en/documents/product-information/xarelto-epar-product-information_en.pdf.

[19] Mueck W, Kubitza D, Becka M (2013). Co-administration of rivaroxaban with drugs that share its elimination pathways: pharmacokinetic effects in healthy subjects. Br J Clin Pharmacol.

